# Habitual sleep durations and subjective sleep quality predict white matter differences in the human brain

**DOI:** 10.1016/j.nbscr.2017.03.001

**Published:** 2017-03-20

**Authors:** Sakh Khalsa, Joanne R. Hale, Aimee Goldstone, Rebecca S. Wilson, Stephen D. Mayhew, Manny Bagary, Andrew P. Bagshaw

**Affiliations:** a Centre for Human Brain Health and School of Psychology, University of Birmingham, Birmingham, UK; b Department of Neuropsychiatry, The Barberry National Centre for Mental Health, Birmingham, UK; c Clinical Physics and Bioengineering, University Hospital Coventry and Warwickshire, Coventry, UK; d Center for Health Sciences, SRI International, Menlo Park, California, USA

## Abstract

Self-imposed short sleep durations are increasingly commonplace in society, and have considerable health and performance implications for individuals. Reduced sleep duration over multiple nights has similar behavioural effects to those observed following acute total sleep deprivation, suggesting that lack of sleep affects brain function cumulatively. A link between habitual sleep patterns and functional connectivity has previously been observed, and the effect of sleep duration on the brain's intrinsic functional architecture may provide a link between sleep status and cognition. However, it is currently not known whether differences in habitual sleep patterns across individuals are related to changes in the brain's white matter, which underlies structural connectivity. In the present study we use diffusion–weighted imaging and a group comparison application of tract based spatial statistics (TBSS) to investigate changes to fractional anisotropy (FA) and mean diffusivity (MD) in relation to sleep duration and quality, hypothesising that white matter metrics would be positively associated with sleep duration and quality. Diffusion weighted imaging data was acquired from a final cohort of 33 (23–29 years, 10 female, mean 25.4 years) participants. Sleep patterns were assessed for a 14 day period using wrist actigraphs and sleep diaries, and subjective sleep quality with the Pittsburgh Sleep Quality Index (PSQI). Median splits based on total sleep time and PSQI were used to create groups of shorter/longer and poorer/better sleepers, whose imaging data was compared using TBSS followed by post-hoc correlation analysis in regions identified as significantly different between the groups**.** There were significant positive correlations between sleep duration and FA in the left orbito-frontal region and the right superior corona radiata, and significant negative correlations between sleep duration and MD in right orbito-frontal white matter and the right inferior longitudinal fasciculus. Improved sleep quality was positively correlated with FA in left caudate nucleus, white matter tracts to the left orbito-frontal region, the left anterior cingulum bundle and the white matter tracts associated with the right operculum and insula, and negatively correlated with MD in left orbito-frontal white matter and the left anterior cingulum bundle. Our findings suggest that reduced cumulative total sleep time (cTST) and poorer subjective sleep quality are associated with subtle white matter micro-architectural changes. The regions we identified as being related to habitual sleep patterns were restricted to the frontal and temporal lobes, and the functions they support are consistent with those which have previously been demonstrated as being affected by short sleep durations (e.g., attention, cognitive control, memory). Examining how inter-individual differences in brain structure are related to habitual sleep patterns could help to shed light on the mechanisms by which sleep habits are associated with brain function, behaviour and cognition, as well as potentially the networks and systems responsible for variations in sleep patterns themselves.

## Highlights

• Habitual sleep duration and quality are associated with changes to DTI metrics.
• Shorter duration and poorer quality link with reduced FA and increased MD.
• Changes are specific to frontal and temporal regions.
• Changes to brain structure could underlie behavioral effects of sleep.

## 1 Introduction

Sleep patterns have been investigated in relation to behaviour and functional connectivity (FC, De Havas et al., 2012; Gujar et al., 2010; Khalsa et al., 2016), but to date there have limited investigations in relation to brain structure. Studies involving patients with chronic insomnia have demonstrated that grey matter in the frontal lobe may be altered with respect to normal sleepers (Altena et al., 2010), while frontal and temporal alterations to cortical thickness (Spira et al. 2016), brain volume (Lo et al., 2014) and white matter intensity (Ramos et al., 2014, Yaffe et al., 2016) have been associated with sleep duration in older adults. Acute sleep deprivation has also been shown to reduce thalamic volume (Liu et al., 2014), although in general the relative impact of acute and long term sleep patterns, as well as the mechanisms underlying them, remain to be understood. These studies suggest that there is a link between sleep duration and brain structure. However, the link between sleep and white matter properties, which form the basis for structural connectivity (SC) and ultimately provide the anatomical substrate for functional interactions, is less well understood.

SC refers to the anatomical links between brain regions, rather than the statistical dependencies between activity time series that are the basis of functional connectivity. It can be characterised non-invasively in humans using diffusion tensor imaging (DTI), with fractional anisotropy (FA) and mean diffusivity (MD), two commonly used metrics to quantify white matter tracts (Beaulieu et al., 2002; Le Bihan, 2003). MD is dependent on the amount of water molecule movement and independent of direction, while FA assesses the directionality of such movement (Le Bihan, 2003). Therefore with reductions in FA a corresponding increase in MD values may often be seen. These measures have been used extensively as markers of white matter microstructural changes in a variety of situations (Alexander et al., 2007), and may be altered by a variety of changes to the underlying white matter, such as increases or decreases in myelination, the extent of coherent alignment of fibres, the presence and orientation of crossing fibres from other bundles etc (Jones et al., 2013), as well as short term plasticity (Engvig et al., 2012) or disease state (e.g., Korgaonkar et al., 2011; Shukla et al., 2011).

In terms of sleep, Rocklage et al. (2009) examined cognitive vulnerability to total sleep deprivation in relation to white matter differences. They found differences in the genu, ascending and longitudinal white matter pathways, with significantly higher FA values in subjects with reduced susceptibility to total sleep deprivation. Elvsashagen et al. (2014) found that a night of total sleep deprivation was associated with widespread FA decreases mainly explained by reductions in axial diffusivity. Piantoni et al. (2013) investigated electroencephalography (EEG) sleep oscillations and DTI metrics and found that individuals with greater spindle power (a phenomenon of N2 and N3 sleep which has been associated with cognitive performance Schabus et al., 2006) demonstrated higher DTI metrics in the corpus callosum and temporal lobe. These observations indicate that the structural correlates of sleep phenomena and even short term alterations to sleep patterns can be investigated with DTI. In combination with the changes to functional connectivity mentioned above, they may also suggest that white matter connectivity and organisation moderates the cognitive effects of sleep deprivation and may affect a person's ability to function effectively when sleep deprived.

In the present study, we use tract based spatial statistics (TBSS, with FDT FSL tool box, Smith et al., 2004) to investigate white matter changes in relation to habitual cumulative sleep time and sleep quality. We chose to measure habitual sleep patterns, using wrist actigraphy recorded over fourteen nights, as these are more representative of a subject's day to day sleep behaviour than experimental sleep deprivation. Our overall aim was to investigate the notion that white matter properties are linked with the long term effects of habitual sleep status and habitual sleep debt. We expected correlations between FA and MD and habitual sleep duration. We also investigated whether subjective habitual sleep quality as measured using the Pittsburgh Sleep Quality Index (PSQI, Buysse et al., 1989) would be related to differences in FA and MD metrics. Furthermore, given the evidence of previous behavioural and functional imaging literature (Belenky et al., 2003, De Havas et al., 2012, Dinges et al., 1997, Gujar et al., 2010, Horne, 1993, Khalsa et al., 2016, Van Dongen et al., 2003), we expected these effects to be most prominent in frontal brain regions.

## 2 Methods and materials

### 2.1 Subjects

DTI and fMRI data were acquired from 38 healthy adults (right handed, 10 female, age 23–29 years, mean age=24.6 years) using a 3 T Philips Achieva MRI scanner at Birmingham University Imaging Centre (BUIC), University of Birmingham. Participants had no history of any neurophysiological, neuropsychological or neurological illness. Written informed consent was obtained from all participants, and the study was approved by the University of Birmingham Ethics Committee. From the original 38 subjects, 5 were subsequently excluded (3 female, mean age 23.4 years) due to actigraphy and diary data demonstrating erratic sleep patterns (for example, settling to sleep at 5am, awakening at 8am and having a 2 h nap at 2 pm), leading to a final cohort of 33 (23–29 years, 10 female, mean 25.4 years) participants.

### 2.2 Sleep patterns and questionnaires

Sleep patterns were assessed for a 14 day period using sleep diaries and wrist actigraphs (Actiwatch2, Philips Respironics Ltd, Cambridge, UK). Actigraphs were set to one minute epochs (which is a medium sensitivity setting). Analysis was performed using Respironics Actiware 5 (Philips, Netherlands) software, with a total sampling rate score of 40 or more was used to identify when the subjects were awake as used in a previous study (Khalsa et al., 2016). Medium or high sampling rate sensitivities have been shown to provide data for the total sleep time (TST) per night in close agreement with polysomnography (PSG, Kushida et al., 2001). Participants also completed the following questionnaires: PSQI, (Buysse et al., 1989), Epworth Sleepiness Scale (ESS, Johns, 1991), Depression, Anxiety and Stress Scale-21 (DASS, Lovibond and Lovibond, 1995) and Karolinska Sleepiness Scale (KSS, Akerstedt and Gillberg, 1990). The questionnaires were administered immediately before or following the scanning session, with the exception of the KSS which was administered verbally immediately upon exiting the scanner. Each of the questionnaires resulted in a single score per subject, while TST was determined from the actigraphy and compared with sleep diary data for consistency (Kushida et al., 2001, Morgenthaler et al., 2007). TST was quantified as cumulative TST (cTST, sum of TST over the entire two week period). Demographic, questionnaire and actigraphic data are summarised in Table 1.

**Table 1:** Summary data for all included subjects: demographics, questionnaires, the mean total habitual sleep time (TST), and cumulative habitual total sleep time (cTST) summed over 14 days.

**Demographics (n=33)**	**Mean**	**SD**
Age (y)	**25.4**	6.27
**Questionnaires**		
Epworth	3.94	.79
Karolinska	1.16	.41
Fatigue	12.36	.98
PSQI	4.5	1.84
Depression	1.42	2.63
Anxiety	1.21	.84
**Actigraphy**		
**Mean TST (h)**	**7.38**	**1.45**
cTST (h)	97.21	8.79

### 2.3 Image acquisition

Subjects underwent a 13 min echo planer DTI scan: TR = 5191 ms, TE = 77 ms, field of view (FOV) = 224×150×224 mm, angulation = 0°, voxel size 2mm isotropic. A total of 75 slices were acquired for b values of b = 0 and b = 1000 mm^2^/s obtained by applying gradients along 61 different diffusion directions. Additionally, a high-resolution (1 mm isotropic) T1-weighted anatomical image was acquired in each subject. Each subject also underwent one resting-state fMRI scan of 12 min duration, during which they were instructed to lie still and relax with eyes open. These data were the subject of a previous publication (Khalsa et al., 2016) and were not analysed further here. All participants confirmed that they remained awake and alert through the scanning session.

### 2.4 Definition of shorter and longer sleepers (cTST)

The DTI data were analysed using TBSS (see below), which requires comparisons between groups. To facilitate this, we split the cohort into groups based on their sleep patterns. The shorter and longer sleeper groups were defined by a median split of the 33 subjects based on the cTST. The 17 subjects with the shortest cTST comprised the shorter sleepers group, and the 16 subjects with the longest cTST made up the longer sleepers group. Table 2 shows the cumulative and mean daily TST for each of the subjects in the two groups.

**Table 2:** cTST and mean daily TST data for all subjects, ordered according to the two groups (shorter and longer sleepers).

**Subject**	**Longer sleepers cTST (h)**	**Mean daily TST (h)**	**Subject**	**Shorter sleepers cTST (h)**	**Mean daily TST (h)**
**1**	99.7	7.12	**1**	65.98	4.71
**2**	99.8	7.13	**2**	73.11	5.22
**3**	100.41	7.17	**3**	84.98	6.07
**4**	100.53	7.18	**4**	85.25	6.08
**5**	102.1	7.29	**5**	85.57	6.11
**6**	103.16	7.37	**6**	85.95	6.13
**7**	103.48	7.39	**7**	86.9	6.2
**8**	103.9	7.42	**8**	89.26	6.37
**9**	103.98	7.43	**9**	89.97	6.42
**10**	105.83	7.55	**10**	91.96	6.56
**11**	106.05	7.57	**11**	92.2	6.58
**12**	108.33	7.73	**12**	93.51	6.67
**13**	108.37	7.74	**13**	94	6.71
**14**	109.15	7.8	**14**	97.5	6.96
**15**	118.48	8.46	**15**	98.03	7
**16**	122.18	8.73	**16**	99.15	7.08
			**17**	99.38	7.09
**Mean**	105.97	7.57		88.33	6.30
**SD**	6.41	0.46		8.77	0.62

### 2.5 Definition of poorer and better sleepers (PSQI)

PSQI global scores for the assessment of sleep quality were used to define subjectively poorer or better sleepers. From the subject group of 33, one subject was excluded due to not filling in the PSQI questionnaire appropriately (responses were vague descriptive words where a tick was required for a specific set of questions). The remaining 32 subjects were split into two groups. The 16 subjects with the lowest PSQI global scores comprised the better sleepers group, and the 16 subjects with the highest PSQI global scores represented the poorer sleepers group. By definition the lower the global PSQI score the better the subjective sleep quality for each subject. PSQI scores for each of the subjects in the two groups are presented in Table 3.

**Table 3:** PSQI scores for better and poorer sleepers.

**Subjects**	**Better sleepers PSQI**	**Subjects**	**Poorer sleepers PSQI**
**1**	1	**1**	5
**2**	1	**2**	5
**3**	2	**3**	5
**4**	2	**4**	5
**5**	2	**5**	5
**6**	2	**6**	6
**7**	2	**7**	6
**8**	3	**8**	6
**9**	3	**9**	6
**10**	3	**10**	6
**11**	3	**11**	7
**12**	3	**12**	7
**13**	4	**13**	7
**14**	4	**14**	8
**15**	4	**15**	8
**16**	4	**16**	10
**Mean**	2.69		6.37
**SD**	1.01		1.41

### 2.6 Tract based spatial statistics (TBSS) analysis

We performed a voxelwise, between group comparison of FA and MD using TBSS (Smith et al., 2006) focusing on a cohort of 33 subjects split into two groups for cTST and 32 subjects split into two groups for subjective sleep quality (PSQI) as described above. TBSS derives estimates of tracks by fitting a tensor model to the raw diffusion-weighted data and assuming that the highest anisotropy is indicative of the centre of white matter tracts. This assumption is not true for all regions. For example where two or more tracts cross, converge or diverge a more complex methodology is required (Smith et al., 2006).

A single FA image from each subject was created using tools in the FDT FSL toolbox (Smith et al., 2004). The original data were corrected for head movement effects and eddy currents (Jenkinson et al., 2002). A brain mask was created using brain extraction tool (BET, Jenkinson et al., 2002) on the non-diffusion weighted image. The diffusion tensor model was fitted using DTIFIT (Part of FSL Tool Box). We then ran the TBSS script for nonlinear registration, aligning all FA images to 1×1×1 mm standard space. The target image used in the registrations was chosen automatically as the most representative of all subjects in the study. This target image was then affine-aligned into 1×1×1 mm MNI152 space (1×1×1 mm resolution was used as the skeletonisation and projection steps are known to work well at 1×1×1 mm resolution, Smith et al., 2006). The FA image for each subject had the nonlinear transform to the target and then the affine transform to MNI152 space applied. This produced a transformation of the original FA image into MNI152 space, hence a standard-space version of the FA image for each subject. These were merged into a single 4D image file. Next, the mean of all FA images was created, and this was used to construct the mean FA skeleton. The last TBSS script was used to threshold the mean FA skeleton at the chosen threshold of 0.2 (Smith et al., 2006) to exclude voxels consisting of grey matter or cerebral spinal fluid.

Voxelwise cross-subject statistics was performed using the randomise tool in FSL which carries out permutation testing (5000 permutations, Nichols and Holmes, 2002). Thresholding was carried out using threshold-free cluster enhancement (TFCE, Smith and Nichols, 2009). The TFCE p-value images produced were fully corrected for multiple comparisons across space to give a significance of p<0.05 to determine which FA voxels were statistically significant between the two groups of subjects. The same procedure was carried out for MD TBSS analysis.

While the groups were required for the TBSS analysis in order to identify regions where FA and MD were related to sleep patterns, the definition of the groups was relatively arbitrary and based on a median split. In practice, we would expect any regions that were identified as being different between the groups to have continuous relationships between FA/MD and sleep patterns across the whole cohort. To confirm this, in addition to the thresholded TBSS images, we extracted mean FA/MD from the regions identified as being significantly different between shorter/longer or poorer/better sleepers and plotted them against cTST/PSQI over all subjects as an additional confirmation of the expected continuous relationships.

## 3 Results

### 3.1 TBSS analysis using cTST

**Fig. 1:**
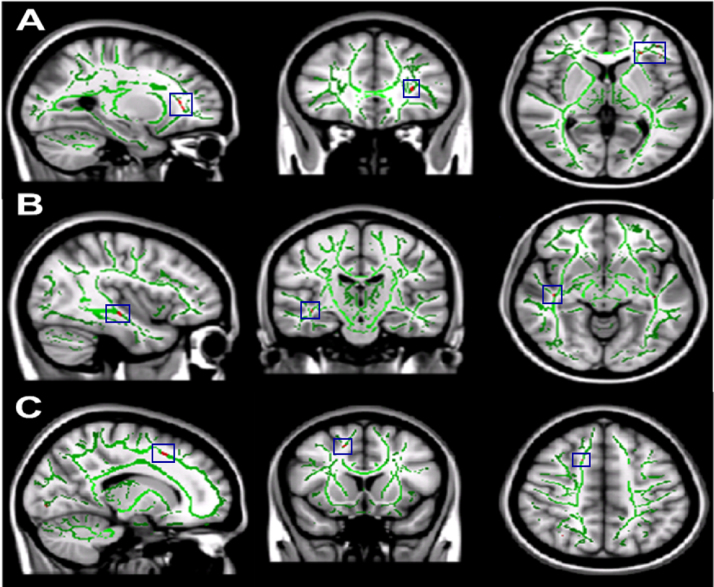
Differences in FA between **longer** and **shorter** sleepers. The mean all sleepers FA skeleton (green) is projected onto the standardised T1 MNI 1mm brain image. The red regions (highlighted in blue boxes) show statistically significant reductions in the mean FA of **shorter** sleepers compared to **longer** sleepers. The significant reductions correspond to (A) the left orbito-frontal region (B) the right inferior fasciculus and (C) the right superior corona radiata. All images shown in radiological convention.

**Fig. 2:**
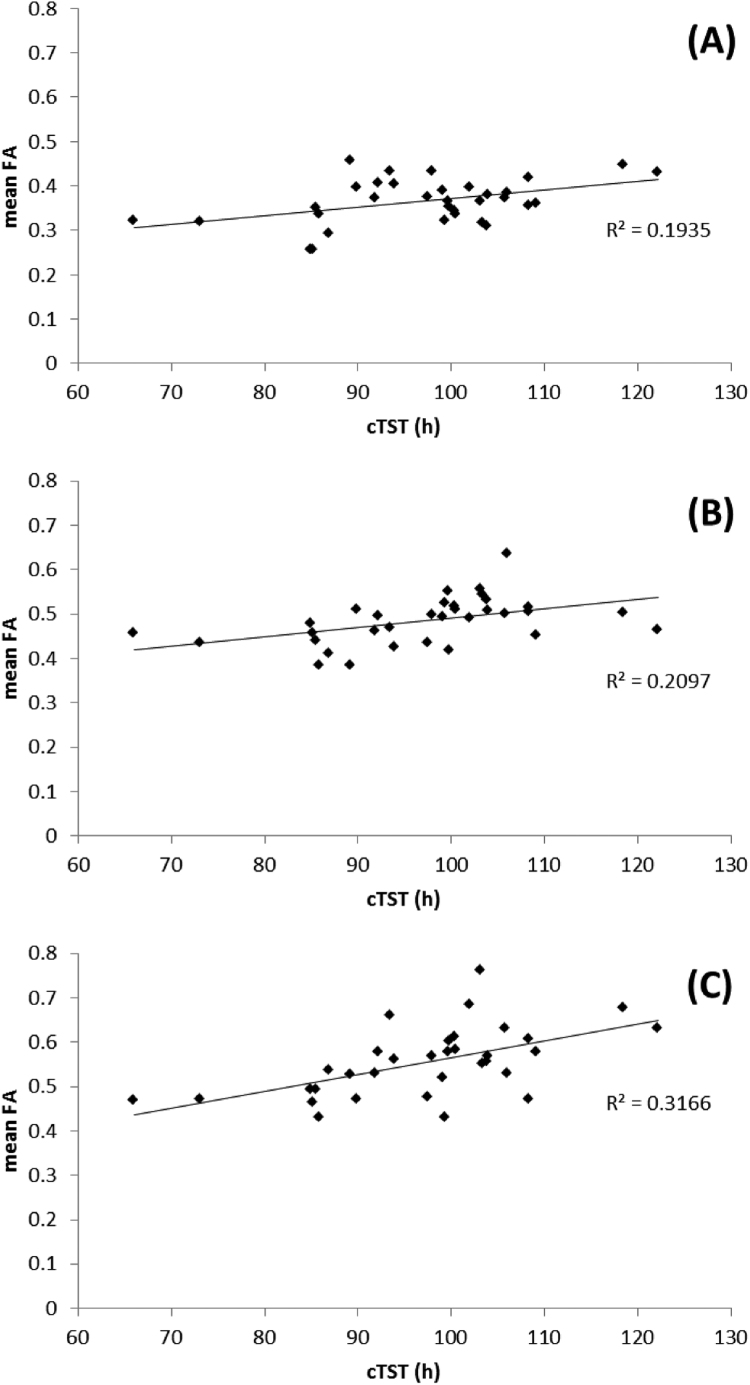
Correlation plots of the relationship between mean FA and cTST over all subjects in regions identified as significantly different between **shorter** and **longer** sleepers: A) left orbito-frontal region, B) right inferior fasciculus and C) right superior corona radiata.

We found statistically significant decreases in mean FA values in shorter sleepers compared to longer sleepers in three brain regions (tracts identified using Mori et al. (2005)): the left orbito-frontal region (MNI [-27, 32, 3]), the right inferior longitudinal fasciculus (MNI [42, -18, -10]) and the right superior corona radiata (MNI [16, 16, 48], see Fig. 1). In order to confirm that these tracts demonstrated a continuous relationship with sleep patterns across the entire cohort, FA was extracted and correlated with cTST (Fig. 2). This demonstrated the expected covariation.

**Fig. 3:**
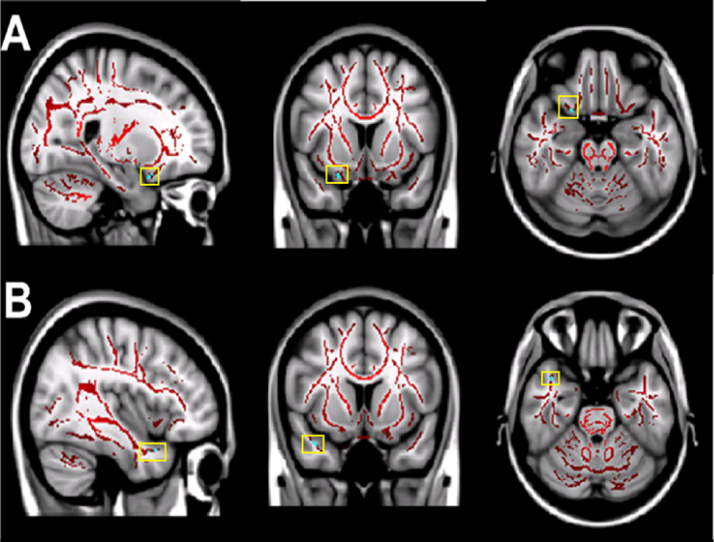
Differences in MD between **longer** and **shorter** sleepers. The mean all sleepers MD skeleton (red) is projected onto the standardised T1 MNI 1mm brain image. The light blue regions (highlighted in yellow boxes) show statistically significant increases in the mean MD of **shorter** sleepers compared to **longer** sleepers. The significant increases correspond to (A) the right orbito-frontal white matter tracts and (B) the right inferior longitudinal fasciculus (temporal pole region). All images shown in radiological convention.

**Fig. 4:**
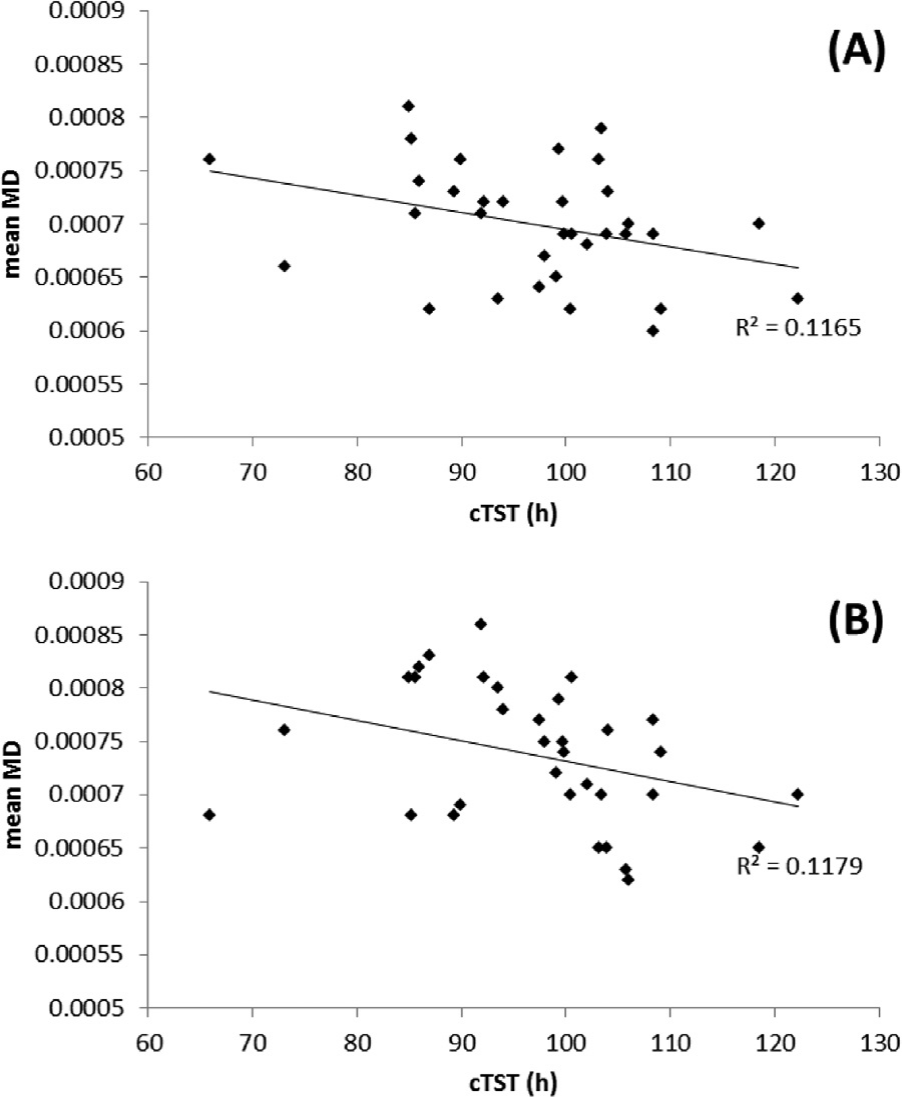
Correlation plots of the relationship between mean FA and cTST over all subjects in regions identified as significantly different between **shorter** and **longer** sleepers:. For A) right orbito-frontal region, B) right inferior fasciculus (temporal pole region).

We also found statistically significant increases in MD values in two brain regions when comparing subjects with shorter cTST against longer cTST: the right orbito-frontal white matter (MNI [19, 19, -17]) and the right inferior longitudinal fasciculus (temporal pole region, MNI [27, -12, -31]). These regions are shown in Fig. 3 and the correlation between MD and cTST in Fig. 4.

#### 3.1.1 TBSS analysis using subject global PSQI scores

**Fig. 5:**
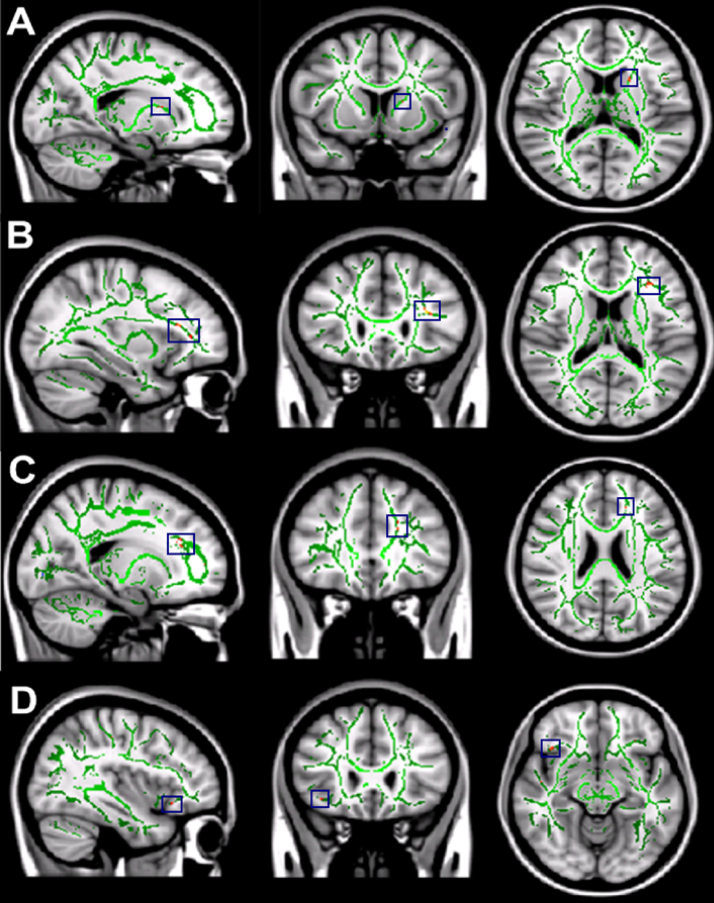
Differences in FA between **poorer** and **better** sleepers measured with the PSQI. The mean all sleepers FA skeleton (green) is projected onto a T1 MNI 1mm standardised brain image. The red regions (highlighted in blue boxes) show statistically significant decreases in the mean FA of **poorer** sleepers compared to **better** sleepers. The significant decreases correspond to (A) the white matter tracts associated with the head of the left caudate nucleus, (B) the white matter tracts associated with the left corona radiata, (C) the left anterior cingulum bundle and (D) the white matter tracts associated with the right operculum and right insula. All images shown in radiological convention.

**Fig. 6:**
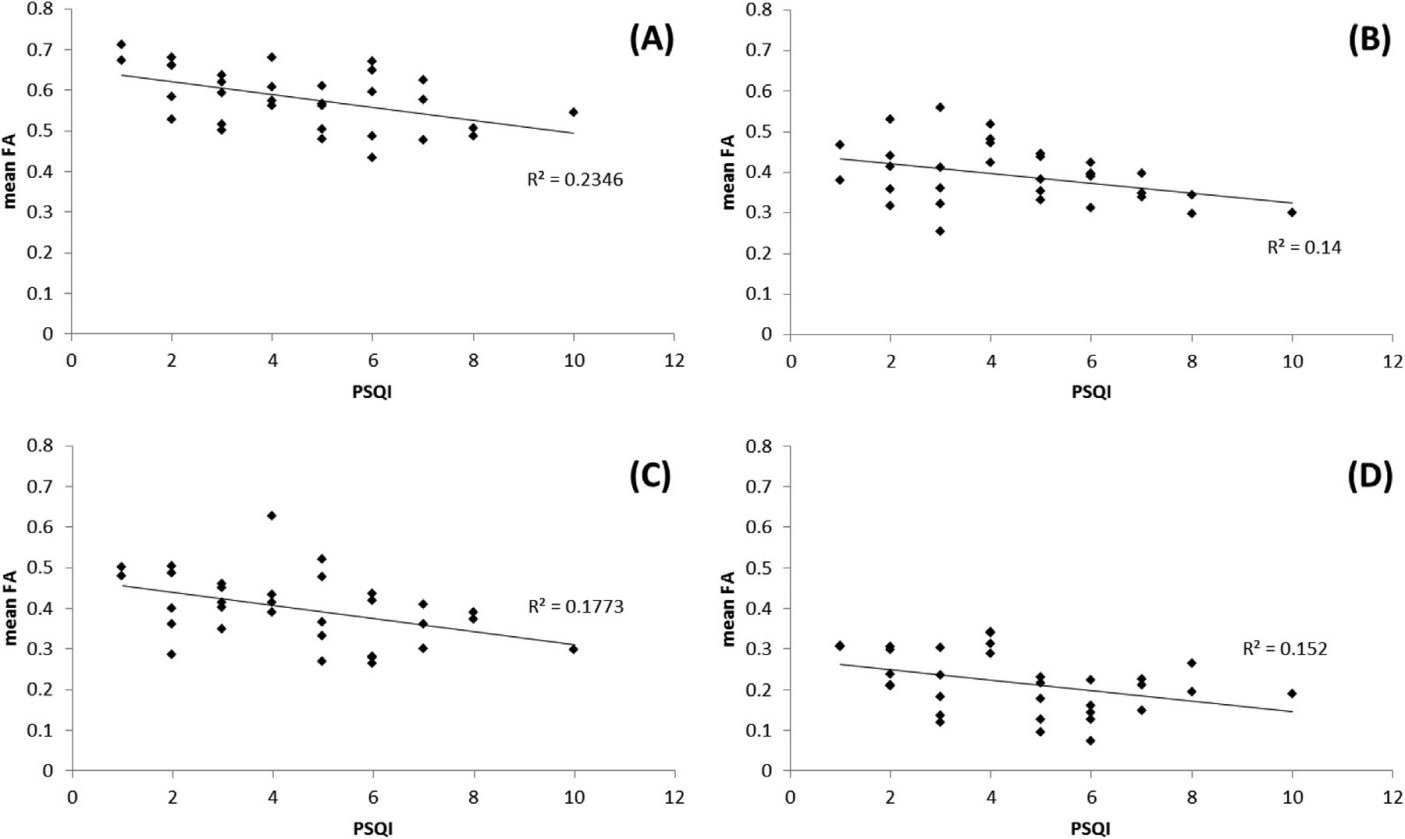
Correlation plots of the relationship between mean FA and PSQI score over all subjects in regions identified as significantly different between **poorer** and **better** sleepers: A) head of the left caudate nucleus, B) left corona radiata, C) left anterior cingulum bundle and D) right insula region.

**Fig. 7:**
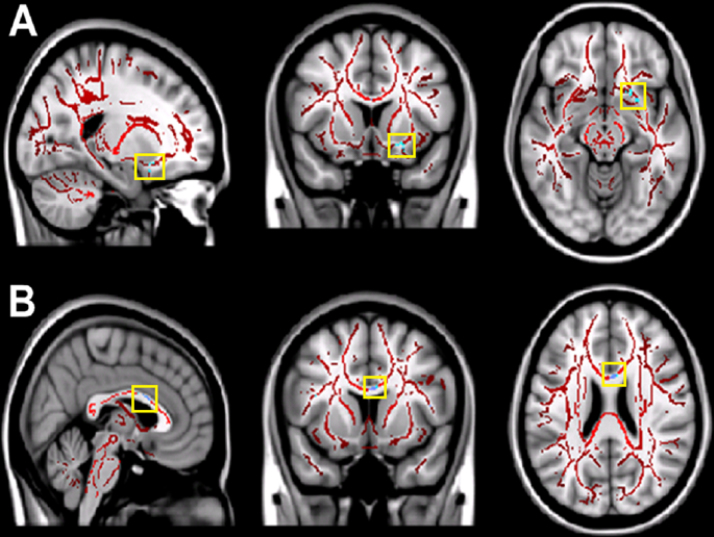
Differences in MD between **poorer** and **better** sleepers. The mean all sleepers MD skeleton (red) is projected onto the standardised T1 MNI 1mm brain image. The light blue regions (highlighted in yellow boxes) show statistically significant increases in the mean MD of **poorer** sleepers compared to **better** sleepers. The significant increases correspond to (A) the left orbito-frontal white matter tracts and (B) the left anterior cingulum bundle. All images shown in radiological convention.

**Fig. 8:**
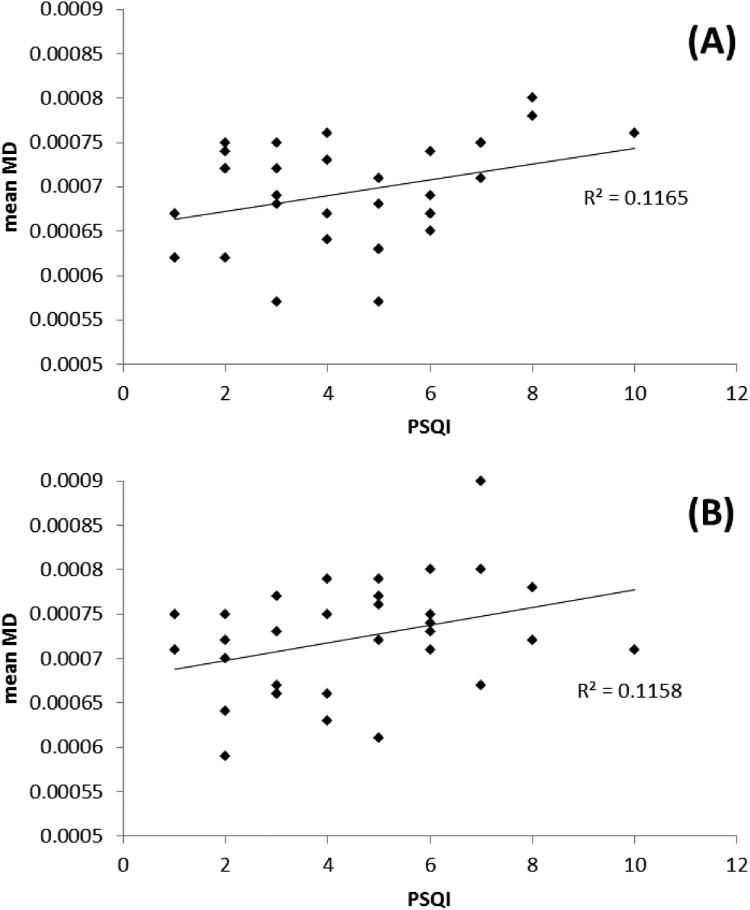
Correlation plots of the relationship between mean MD and PSQI score over all subjects in regions identified as significantly different between **poorer** and **better** sleepers: A) left orbito-frontal region, B) left anterior cingulum.

We found statistically significant differences in mean FA and MD values when comparing DTI metrics of subjects with poorer subjective sleep quality (high PSQI global scores) against subjects with better subjective sleep quality (low PSQI global scores). Significant decreases in mean FA were found in four white matter brain regions for the subjects with poorer subjective sleep quality relative to those with better sleep quality: white matter tracts to the head of the left caudate nucleus (MNI [-19, 18, 11]); white matter tracts to the left orbito-frontal region (MNI [-34, 29, 13]), the left anterior cingulum bundle (MNI [-17, 23, 24]) and the white matter tracts associated with the right operculum and insula (MNI [39, 24, -12], Fig. 5. In each of these regions, there was a continuous relationship between FA and sleep quality (Fig. 6). Significantly higher mean MD values were found for the left orbito-frontal white matter (MNI [-20, 29, -6]) and the left anterior cingulum bundle (MNI [-6, 18, 18], Fig. 7), in both cases demonstrating a continuous relationship with PSQI across the whole cohort (Fig. 8).

## 4 Discussion

We used TBSS to investigate whole brain changes in white matter architecture in relation to habitual sleep patterns, as quantified by cTST, and sleep quality, as quantified by the PSQI. In both cases, we were able to identify specific white matter tracts where differences in FA or MD were related to sleep metrics, demonstrating that objective and subjective measures of habitual sleep are associated with the brain's white matter structure.

The differences when comparing the generally poorer sleep group (i.e., shorter cTST or higher PSQI) with the generally better sleep group (i.e., longer cTST or lower PSQI) always indicated lower FA and/or higher MD. These were confirmed as continuous relationships across all subjects, as would be expected for a cohort of healthy sleepers. While many physical and physiological factors contribute to the quantification of these DTI measures (Jones et al., 2013), both reductions in FA and increases in MD have generally been linked with reductions in behavioural and cognitive performance (see below). Our observations of these changes to brain structure in relation to sleep would therefore be consistent with the considerable literature on the behavioural and cognitive effects of acute or chronic sleep deprivation, which does not point towards improved performance with poor sleep. The differences in white matter properties that we see could therefore form the underlying substrate of the behavioural and cognitive effects of poor sleep patterns.

While we assessed sleep duration only over a two week period, and sleep quality as a single measure, inter-individual differences in sleep patterns are generally stable in the short and long term (Briscoe et al., 2014, Gaines et al., 2015, Otte et al., 2011) and may have an underlying genetic basis (Barclay and Gregory, 2013). In addition, individuals with shorter habitual sleep durations show evidence of a higher sleep debt than those with habitually longer sleep duration (Klerman and Dijk, 2005). Overall, these studies suggest that the inter-individual differences we characterised are representative of stable, habitual variations in sleep patterns over a longer period than we measured, and that the shorter sleepers may be experiencing cumulative sleep debt. Future longitudinal and/or interventional studies with longer actigraphic recording periods will be needed to confirm the causality of the relationship between sleep patterns and brain structure.

We found a significant reduction in mean FA, and a significant increase in MD, for the shorter sleepers compared to the longer sleepers in the left orbito-frontal region, right superior corona radiata and right inferior longitudinal fasciculus. The white matter changes observed in the orbito-frontal regions are consistent with regions known to be affected by sleep deprivation and habitual sleep durations in functional MRI studies (Bosch et al., 2013, De Havas et al., 2012, Gujar et al., 2010, Khalsa et al., 2016, Tomasi et al., 2009, Verweij et al., 2014). With respect to the corona radiata, there is a considerable body of literature which has identified alterations to their structural properties and linked them with behavioural performance deficits, particularly in relation to attention and cognitive control (Durston et al., 2006; Leite et al., 2013; Liston et al., 2011; Niogi et al., 2010; Thillainadesan et al., 2012). FA decreases in the anterior corona radiata also suggest possible disruption of thalamocortical connective relays of the frontal cortex (Niogi et al., 2010). Corona radiata alterations in subjects with short sleep compared to those with long sleep would therefore be consistent with an underlying structural basis for the observed behavioural effects of short sleep durations, particularly in relation to cognitive control and attention.

A negative correlation between FA and sleep duration was also observed in the right inferior longitudinal fasciculus, which is known to have numerous projections to the superior temporal regions and also to the long fibres of the posterior cingulum bundle (Catani et al., 2003). Such reductions may suggest subtle disruption to relays from posterior cingulate/parietal and other cortical areas to the temporal lobe which may in turn affect functional interactions between these cortical regions resulting in subtle changes such as memory impairment (Hayes et al., 2012). Haller et al. (2013) reported group level decreases in FA using TBSS analysis for the right inferior longitudinal fasciculus in patients with mild cognitive impairment, while Ortibus et al. (2012) suggested an association between reduced FA in the inferior longitudinal fasciculus and object recognition deficits in children with visual impairment compared to normal controls. Studies investigating the effect of sleep deprivation on object recognition and memory consolidation in rodents (Palchykova et al., 2006a, Palchykova et al., 2006b) and in humans (Chee et al., 2010) have shown impairment in object recognition and memory for sleep deprived subjects compared to normal controls. Overall, it is plausible that changes in the structure of tracts such as the orbito-frontal regions, superior corona radiata and the inferior longitudinal fasciculus may contribute to attentional and other cognitive impairments which are commonly seen as a function of sleep deprivation and poor sleep quality (Alhola et al., 2007; Babkoff et al., 2005; Banks and Dinges, 2007; Belenky et al., 2003; Dinges et al., 1997; Van Dongen et al., 2003).

We also demonstrated significant, regionally specific relationships between DTI metrics and subjective sleep quality. Subjects who had poorer sleep quality had reduced FA in white matter regions associated with the left caudate, left orbito-frontal region, left anterior cingulum bundle and the right insula compared to subjectively better sleepers. It has been suggested from functional MRI studies that the caudate nucleus is linked to neural networks involved in the regulation of executive function, sleep and arousal (Stoffers et al., 2014). Lesions to the caudate in animal studies (Villablanca et al., 1976) induced restlessness and hyper-arousal which indicate failing inhibitory modulation of sensory inputs. Therefore from our findings we can postulate that poorer subjective sleepers may demonstrate subtle reductions in inhibitory modulation compared to better subjective sleepers due to the comparatively reduced DTI parameters in the orbito-frontal regions and caudate white matter. This may suggest a subtle state of hyper-arousal in poor subjective sleepers, and may partly explain the impairment of left caudate recruitment during executive function (Stoffers et al., 2014), and the state of hyper-arousal which has been reported (Bonnet and Arand, 2010) in subjects with sleep pathology such as insomnia.

We found significant reductions in FA in the left anterior cingulum bundle and white matter associated with the right insula, as well as increases in MD in left orbito-frontal white matter and the left anterior cingulum. These regions may suggest a connection with the corresponding cortical areas that form part of the salience network (Seeley et al., 2010). The right anterior insula is thought to act as a control switch between the central executive network and the default mode network (Sridharan et al., 2008) and is involved in the brain's attention system (Eckert et al., 2009). A recent study has shown the functional connectivity between the right insula and the mesial prefrontal cortex to co-vary with cTST (Khalsa et al., 2016), suggesting a link between saliency and quantitative measures of habitual sleep status. The current study extends these findings, and future studies will be needed to investigate more explicitly whether alterations to functional and structural connectivity mediate the relationship between short sleep and saliency and attention (Belenky et al., 2003, Dinges et al., 1997, Van Dongen et al., 2003).

In conclusion, our findings demonstrate regionally-specific relationships between habitual cTST and subjective sleep quality and white matter micro-architecture. Given that white matter forms the structural skeleton for functional interactions between brain regions which ultimately underpin behaviour and cognition, it is likely that the links we see between white matter and sleep patterns would have functional and behavioural consequences. The regions we identified as being related to habitual sleep patterns were restricted to the frontal and temporal lobes, and the functions they support are consistent with those which have previously been demonstrated as being affected by short sleep durations (e.g., attention, memory, cognitive control). Examining how inter-individual differences in brain structure are related to habitual sleep patterns could help to shed light on the mechanisms by which sleep habits are associated with brain function, behaviour and cognition, as well as potentially the networks and systems responsible for variations in sleep patterns themselves.

## Disclosure statement

This was not an industry-supported study. This work was supported by the UK Engineering and Physical Sciences Research Council (grant number EP/J002909/1). Stephen D. Mayhew was funded by an EPSRC Fellowship (grant number EP/I022325/1) and a Birmingham University Fellowship. Manny Bagary is supported by UCB, Eisai, and Cyberonics but the work reported in this article is not related to those relationships. The other authors have indicated no financial conflicts of interest.

## References

[bib1] Akerstedt T., Gillberg M. (1990). Subjective and objective sleepiness in the active individual. Int. J. Neurosci..

[bib2] Alexander A.L., Lee J.E., Lazar M., Field A.S. (2007). Diffusion tensor imaging of the brain. Neurotherapeutics.

[bib3] Alhola P., Polo-Kantola P. (2007). Sleep deprivation: impact on cognitive performance. Neuropsychiatr. Dis. Treat..

[bib4] Altena E., Vrenken H., Van Der, Werf Y.D., Van, den Heuvel O.A., Van Someren E.J.W. (2010). Reduced orbitofrontal and parietal grey matter in chronic insomnia: a voxel-based morphometric study. Biol. Psychol..

[bib5] Babkoff H., Zukerman G., Folstick L. (2005). Effect of the diurnal rhythm and 24 h of sleep deprivation on dichotic temporal order judgement. Sleep.

[bib6] Banks S., Dinges D.F. (2007). Behavioral and physiological consequences of sleep restriction. J. Clin. Sleep Med..

[bib7] Barclay N.L., Gregory A.M. (2013). Quantitative genetic research on sleep: a review of normal sleep, sleep disturbances and associated emotional, behavioural and health-related difficulties. Sleep Med. Rev..

[bib8] Beaulieu C. (2002). The basis of anisotropic water diffusion in the nervous system–a technical review. NMR Biomed..

[bib9] Belenky G., Wesensten N.J., Thorne D.R., Thomas M.L., Sing H.C., Redmond D.P., Balkin T.J. (2003). Patterns of performance degradation and restoration during sleep restriction and subsequent recovery: a sleep dose‐response study. Sleep.

[bib10] Bonnet M.H., Arand D.L. (2010). Hyperarousal and insomnia: state of the science. Sleep. Med. Rev..

[bib11] Bosch O.G., Rihm J.S., Scheidegger M., Landolt H.P., Stampfli P., Brakowski J., Esposito F., Rasch B., Seifritz E. (2013). Sleep deprivation increases dorsal nexus connectivity to the dorsolateral prefrontal cortex in humans. Proc. Natl. Acad. Sci. USA.

[bib12] Briscoe S., Hardy E., Pengo M.F., Kosky C., Williams A.J., Hart N., Steir J. (2014). Comparison of 7 versus 14 days wrist actigraphy monitoring in a sleep disorders clinic population. Chronobiol. Int..

[bib13] Buysse D.J., Reynolds C.F., Monk T.H., Berman S.R., Kupfer D.J. (1989). The Pittsburgh Sleep Quality Index: a new instrument for psychiatric practice and research. Psychol. Res..

[bib14] Catani M., Jones D.K., Donato R. (2003). Occipito‐temporal connections in the human brain. Brain.

[bib15] Chee M.W., Tan J.C., Parimal S., Zagorodnov V. (2010). Sleep deprivation and its effects on object-selective attention. Neuroimage.

[bib16] De Havas J.A., Parimal S., Soon C.S., Chee M.W. (2012). Sleep deprivation reduces default mode network connectivity and anti-correlation during rest and task performance. Neuroimage.

[bib17] Dinges D.F., Pack F., Williams K., Gillen K.A., Powel J.W., Ott G.E., Pack A.I. (1997). Cumulative sleepiness, mood disturbance and psychomotor vigilance performance decrements during a week of sleep restricted to 4-5 h per night. Sleep.

[bib18] Durston S., Casey B.J. (2006). What have we learned about cognitive development from neuroimaging?. Neuropsychologia.

[bib19] Eckert M.A., Menon V., Walzak A., Ahlstrom J., Denslow S., Horowitz A., Dubno J.R. (2009). At the heart of the ventral attention system: the right anterior insula. Hum. Brain Mapp..

[bib20] Elvsashagen T., Norbom L.B., Pedersen P.O., Quraishi S.H., Bjornerud A., Malt U.F., Groote I.R., Westlye L.T. (2014). Widespread changes in white matter microarchitecture after a day of waking and sleep deprivation. PloS One.

[bib21] Engvig A., Fjell A.M., Westlye L.T., Moberget T., Sundseth Ø., Larsen V.A., Walhovd K.B. (2012). Memory training impacts short‐term changes in aging white matter: a longitudinal diffusion tensor imaging study. Hum. Brain Mapp..

[bib22] Gaines J., Vgontzas A.N., Fernandez-Mendoza J., Basta M., Pejovic S., He F., Bixler E.O. (2015). Short- and long-term stability in insomniacs and healthy controls. Sleep.

[bib23] Gujar N., Yoo S.S., Hu P., Walker M.P. (2010). The unrested resting brain: sleep deprivation alters activity within the default-mode network. J. Cogn. Neurosci..

[bib24] Haller S., Missonnier P., Herrmann F.R., Rodriguez C., Deiber M.P., Nguyen D., Giannakopoulos P. (2013). Individual classification of mild cognitive impairment subtypes by support vector machine analysis of white matter DTI. Am. J. Neuroradiol..

[bib25] Hayes S.M., Salat D.H., Verfaellie M. (2012). Default network connectivity in medial temporal lobe amnesia. J. Neurosci..

[bib26] Horne J.A. (1993). Human sleep, sleep loss and behavior: implications for the prefrontal cortex and psychiatric disorder. Br. J. Psychiatry.

[bib27] Jenkinson M., Bannister P., Brady M., Smith S. (2002). Improved optimization for the robust and accurate linear registration and motion correction of brain images. Neuroimage.

[bib28] Johns M.W. (1991). A new method for measuring daytime sleepiness: the Epworth sleepiness scale. Sleep.

[bib29] Jones D.K., Knosche T.R., Turner R. (2013). White matter integrity, fiber count and other fallacies: the do's and don’ts of diffusion MRI. NeuroImage.

[bib30] Khalsa S., Mayhew S.D., Przezdzik I., Wilson R., Hale J., Goldstone A., Bagary M., Bagshaw A.P. (2016). Variability in cumulative habitual sleep duration predicts waking functional connectivity. Sleep.

[bib31] Klerman E.B., Dijk D.J. (2005). Interindividual variation in sleep duration and its association with sleep debt in young adults. Sleep.

[bib32] Korgaonkar M.S., Grieve S.M., Koslow S.H., Gabrieli J.D., Gordon E., Williams L.M. (2011). Loss of white matter integrity in major depressive disorder: evidence using tract‐based spatial statistical analysis of diffusion tensor imaging. Hum. Brain Mapp..

[bib33] Kushida C.A., Chang A., Gadkary C., Guilleminault C., Carrillo O., Dement W.C. (2001). Comparison of actigraphic, polysomnographic, and subjective assessment of sleep parameters in sleep-disordered patients. Sleep.

[bib34] Le Bihan D. (2003). Looking into the functional architecture of the brain with diffusion MRI. Nat. Rev. Neurosci..

[bib35] Leite S.C.B., Corrêa D.G., Doring T.M., Kubo T.T.A., Netto T.M., Ferracini R., Ventura N., Bahia P.R.V., Gasparetto E.L. (2013). Diffusion tensor MRI evaluation of the corona radiata, cingulate gyri, and corpus callosum in HIV patients. J. Magn. Reson. Imag..

[bib36] Liston C., Cohen M.M., Teslovich T., Levenson D., Casey B.J. (2011). Atypical prefrontal connectivity in attention-deficit/hyperactivity disorder: pathway to disease or pathological end point?. Biol. Psychiatry.

[bib37] Liu C., Kong X., Liud X., Zhou R., Wu B. (2014). Long-term total sleep deprivation reduces thalamic gray matter volume in healthy men. NeuroReport.

[bib38] Lo J.C., Loh K.K., Zheng H., Sim S.K., Chee M.K. (2014). Sleep duration and age-related changes in brain structure and cognitive performance. Sleep.

[bib39] Lovibond P.F., Lovibond S.H. (1995). Manual for the Depression Anxiety Stress Scales (DASS).

[bib40] Morgenthaler T.I., Lee-Chiong T., Alessi C., Friedman L., Aurora R.N., Boehlecke B., Brown T., Chesson A.L., Kapur V., Maganti R., Owens J., Pancer J., Swick T.J., Zak R. (2007). Practice parameters for the clinical evaluation and treatment of circadian rhythm sleep disorders. Sleep.

[bib41] Mori S., Oishi K., Faria A., van Zijl C.M. (2011). MRI Atlas of Human white matter.

[bib42] Nichols T.E., Holmes A.P. (2002). Nonparametric permutation tests for functional neuroimaging: a primer with examples. Hum Brain Mapp..

[bib43] Niogi S., Mukherjee P., Ghajar J., McCandliss B.D. (2010). Individual differences in distinct components of attention are linked to anatomical variations in distinct white matter tracts. Front. Neuroanat..

[bib44] Ortibus E., Verhoeven J., Sunaert S., Casteels I., De Cock P., Lagae L. (2012). Integrity of the inferior longitudinal fasciculus and impaired object recognition in children: a diffusion tensor imaging study. Dev. Med. Child Neurol..

[bib45] Otte J.L., Payne J.K., Carpenter J.S. (2011). Nighttime variability in wrist actigraphy. J. Nurs. Meas..

[bib46] Palchykova S., Crestani F., Meerlo P., Tobler I. (2006). Sleep deprivation and daily torpor impair object recognition in Djungarian hamsters. Physiol. Behav..

[bib47] Palchykova S., Winsky-Sommerer R., Meerlo P., Dürr R., Tobler I. (2006). Sleep deprivation impairs object recognition in mice. Neurobiol. Learn Mem..

[bib48] Piantoni G., Poil S.S., Linkenkaer-Hansen K., Verweij I.M., Ramautar J.R., Van Someren E.J., Van Der Werf Y.D. (2013). Individual differences in white matter diffusion affect sleep oscillations. J. Neurosci..

[bib49] Ramos A.R., Dong C., Rundek T., Elkind M.S., Boden-Albala B., Sacco R.L., Wright C.B. (2014). Sleep duration is associated with white matter hyperintensity volume in older adults: the Northern Manhattan Study. J. Sleep Res..

[bib50] Rocklage M., Williams V., Pacheco J., Schnyer D.M. (2009). White matter differences predict cognitive vulnerability to sleep deprivation. Sleep.

[bib51] Schabus M., Hödlmoser K., Gruber G., Sauter C., Anderer P., Klösch G., Parapatics S., Saletu B., Klimesch W., Zeitlhofer J. (2006). Sleep spindle‐related activity in the human EEG and its relation to general cognitive and learning abilities. Eur. J. Neurosci..

[bib52] Seeley W.W., Menon V., Schatzberg A.F., Keller J., Glover G.H., Kenna H., Reiss A.L., Greicius M.D. (2010). Dissociable intrinsic connectivity networks for salience processing and executive control. J. Neurosci..

[bib53] Shukla D.K., Keehn B., Müller R.A. (2011). Tract‐specific analyses of diffusion tensor imaging show widespread white matter compromise in autism spectrum disorder. J. Child Psychol. Psychiatry.

[bib54] Smith S.M., Jenkinson M., Johansen-Berg H., Rueckert D., Nichols T.E., Mackay C.E., Watkins K.E., Ciccarelli O., Cader Z.M., Matthews P.M., Behrens T.E.J. (2006). Tract-based spatial statistics: voxelwise analysis of multi-subject diffusion data. NeuroImage.

[bib55] Smith S.M., Jenkinson M., Woolrich M.W., Beckmann C.F., Behrens T.E.J., Johansen-Berg H., Bannister P.R. (2004). Advances in functional and structural MR image analysis and implementation as FSL. Neuroimage.

[bib56] Smith S.M., Nichols T.E. (2009). Threshold-free cluster enhancement: addressing problems of smoothing, threshold dependence and localisation in cluster inference. Neuroimage.

[bib57] Spira A.P., Gonzalez C.E., Venkatraman V.K., Wu M.N., Pacheco J., Simonsick E.M., Ferrucci L., Resnick S.M. (2016). Sleep duration and subsequent cortical thinning in cognitively normal older adults. Sleep.

[bib58] Sridharan D., Levitin D.J., Menon V.A. (2008). Critical role for the right fronto-insular cortex in switching between central-executive and default-mode networks. Proc. Natl. Acad. Sci. USA.

[bib59] Stoffers D., Altena E., van der Werf Y.D., Sanz-Arigita E.J., Voorn T.A., Astill R.G., Strijers R.L.M., Waterman D., Van Someren E.J.W. (2014). The caudate: a key node in the neuronal network imbalance of insomnia?. Brain.

[bib60] Thillainadesan S., Wen W., Zhuang L., Crawford J., Kochan N., Reppermund S., Sachdev P. (2012). Changes in mild cognitive impairment and its subtypes as seen on diffusion tensor imaging. Int. Psychogeriatr..

[bib61] Tomasi D., Wang R.L., Telang F., Boronikolas V., Jayne M.C., Wang G.-J., Wang J., Fowler S., Volkow N.D. (2009). Impairment of attentional networks after 1 night of sleep deprivation. Cereb. Cortex.

[bib62] Van Dongen H.P., Maislin G., Mullington J.M., Dinges D.F. (2003). The cumulative cost of additional wakefulness: dose-response effects on neurobehavioral functions and sleep physiology from chronic sleep restriction and total sleep deprivation. Sleep.

[bib63] Verweij I.M., Romeijn N., Smit D.J., Pantoni G., Van Someren E.J., van der Werf Y.D. (2014). Sleep deprivation leads to a loss of functional connectivity in frontal brain regions. BMC Neurosci..

[bib64] Villablanca J.R., Marcus R.J., Olmstead C.E. (1976). Effects of caudate nuclei or frontal cortical abilations in cats.II. Sleep-wakefulness, EEG, and motor activity. Exp. Neurol..

[bib65] Yaffe K., Nasrallah I., Hoang T.D., Lauderdale D.S., Kuntson K.L., Carnethon M.R., Launer L.J., Lewis C.E., Sidney S. (2016). Sleep duration and white matter quality in middle-aged adults. Sleep.

